# A comprehensive evaluation of epidemiological evidence on processed meat intake in relation to cancer, cardiovascular, metabolic diseases and all-cause mortality: an umbrella review

**DOI:** 10.3389/fpubh.2026.1763155

**Published:** 2026-04-28

**Authors:** Jiajia Ren, Mihray Ablimit, Tuohatijiang Maimaitiaili, ZainaFuguli Tuoheti, Zhang Jing, Dongliang Jiao, HongBao Xue, Nuramatjan Ablat

**Affiliations:** 1 Graduate School, Bengbu Medical University, Bengbu, Anhui, China; 2 Campus Hospital of Bengbu Medical University, Bengbu, China; 3 Department of Pharmacy, The First People's Hospital of Kashi Prefecture, Kashi Xinjiang, China; 4 Xinjiang Uygur Autonomous Region Kuche City Biyixibage Township Health Center, Kuche, Xinjiang, China; 5 School of Mental Health, Bengbu Medical University, Bengbu, Anhui, China; 6 School of Pharmacy, Bengbu Medical University, Bengbu, Anhui, China

**Keywords:** cancer, cardiovascular diseases, endocrine diseases, meta-analysis, processed meat, umbrella review

## Abstract

**Objective:**

This study aims to provide a comprehensive overview of existing systematic reviews and meta-analyses, systematically summarizing the current evidence on the topic and exploring the relationship between processed meat (PM) consumption and various health outcomes.

**Methods:**

A systematic search was conducted in PubMed, Embase, and Web of Science databases for systematic reviews and meta-analyses examining the relationship between PM consumption and health outcomes from the inception of each database until May 2025. This is a descriptive umbrella review that summarizes existing systematic reviews and meta-analyses without conducting further quantitative re-analysis of summary estimates using random- or fixed-effects models or prediction intervals. A total of 34 articles and 54 meta-analyses were included in the review. Use the validated AMSTAR2 tool to assess the methodological quality of studies included in the meta-analysis, and use GRADE to assign the level of evidence quality.

**Results:**

The key dose-response results reported below were selected based on their high public health relevance and stable effect estimates across the included meta-analyses. Consumption of PM was found to be associated with higher risks of various cancers, cardiovascular diseases, metabolic disorders, and all-cause mortality. Dose-response analysis revealed that an increase in PM consumption of 50 g/day was associated with a 72% higher risk of gastric cancer, 17% higher risk of colorectal cancer, 4% higher risk of prostate cancer, and an 8% higher risk of chronic obstructive pulmonary disease (COPD). All the above dose-response associations are based on low to very low certainty evidence as assessed by the GRADE system.

**Conclusion:**

Most studies suggest that PM consumption is associated with diseases such as cancer, cardiovascular diseases, and metabolic disorders, but the certainty of evidence for most outcomes is rated as low or very low by the GRADE system. Based on the report from the International Agency for Research on Cancer (WHO-IARC) and the World Cancer Research Fund (WCRF), no level of PM intake can be confidently considered safe for the prevention of chronic diseases such as colorectal cancer. Future research should include more well-designed prospective studies and randomized controlled trials to further explore the associations between PM consumption and various health outcomes.

## Introduction

Processed meat (PM) products refer to meats that have been treated through methods such as curing, drying, fermenting, smoking, or other processes to enhance flavor or facilitate preservation. Most PM products contain pork or beef, and may also include poultry, offal, or meat by-products, including blood (1). Processed red meat (PRM) is a subtype of PM, specifically referring to preserved meats derived from mammalian muscle (e.g., beef, pork, lamb). Unprocessed red meat (URM) denotes fresh mammalian muscle meat without any curing, smoking, brining, or chemical preservative treatment (2). With economic development and lifestyle changes, both developed and developing countries continue to experience an increasing demand for PM products (3). A global survey of animal-source food consumption across 185 countries found that the average daily intake of PM worldwide increased by 152.8% from 1990 to 2018, reaching 17 g/day. Among these countries, Germany, Russia, the Philippines, and Brazil had the highest consumption levels, while Bangladesh, India, Tanzania, and Turkey had the lowest. In 32 out of the 185 countries, the average intake of PM was at least one serving per day (50 grams per serving) (4). In the United States, consumers' average consumption of PM was 182 g/ week from 1990 to 2000 and 187 g/week from 2015 to 2016 (5). Italian consumers had an average weekly consumption of PM of 245 grams during the period of 2005 to 2006 (6). PM, which is widely consumed in human diets worldwide, has been increasingly associated with adverse health outcomes in recent years. These primarily include cardiovascular diseases (7), cancers (8, 9), all-cause mortality (10), and type 2 diabetes (11). Due to the accumulating evidence of the carcinogenicity of PM, major domestic and international organizations currently recommend consumers to limit their intake of PM to a certain level. For instance, the US Dietary Guidelines for Americans from 2015 to 2020 suggested limiting the consumption of PM to about one serving per week (12). On October 26, 2015, the International Agency for Research on Cancer (IARC), affiliated with the World Health Organization in Lyon, France, classified PM as a Group 1 carcinogen (1).

Despite the publication of numerous meta-analyses of observational studies and randomized controlled trials in recent years, focusing on the association between PM consumption and a range of health outcomes, drawing clear conclusions remains challenging due to flaws in study design, variations in the measurement of intake, inconsistent results, and differing definitions of exposure. Therefore, before formulating detailed health policies related to PM, a comprehensive assessment of the quality of existing evidence on the associations between PM consumption and all health outcomes is necessary. Therefore, this study collected systematic reviews and meta-analyses on the consumption of PM and various health outcomes, and conducted an umbrella review to quantitatively assess the relationship between PM intake and multiple health outcomes, providing scientific evidence for the rationalization of dietary nutrition for consumers.

## Methods

### Overview of methods

Umbrella review, also known as systematic review of reviews, is a comprehensive research method that summarizes all systematic reviews and meta-analyses of evidence on a specific research topic and re-evaluates them. It assesses the strength of evidence and risk of bias in published systematic reviews and meta-analyses, thus yielding more reliable conclusions. The umbrella review has been registered in PROSPERO (CRD42022310748). This study is designed as a descriptive umbrella review; no further quantitative re-analysis (e.g., random/fixed-effects models, prediction intervals) of the summary estimates from the included meta-analyses was performed, in line with the pre-specified study protocol. This study is designed as a descriptive umbrella review; no further quantitative re-analysis of summary estimates from the included meta-analyses was performed due to high heterogeneity and unavailability of individual participant data.

### Literature search

We conducted a systematic search of observational or experimental studies evaluating processed meat intake in the PubMed, Embase, and Web of Science databases, from inception to May 2025. The search strategy involved combining medical subject heading terms, keywords, and variants of text terms related to processed meat to retrieve systematic reviews and meta-analyses: (“processed meat”) and (“systematic review” or “meta-analysis”). Taking PubMed as an example, the specific search strategy is outlined below:

#1 “processed meat” [MeSH Terms] OR “processed meat products^*^” [Title/ abstract] OR “Pre-processed meat^*^” [Title/ abstract] OR “ Processing of meat products^*^” [Title/ abstract]

#2 “systematic review” [MeSH Terms] OR “system assessment^*^” [Title/ abstract] OR “ system evaluation^*^” [Title/ abstract] OR “Meta-analysis^*^” [Title/ abstract] OR “Meta analysis^*^” [Title/ abstract] OR “Meta^*^” [Title/ abstract]

#3 #1 AND #2

Two researchers independently screened titles and abstracts, and selected articles that met the criteria by reading the full text. In cases of disagreement between the two researchers in the selection process, a third researcher was consulted to resolve the dispute.

### Eligibility criteria

#### Inclusion criteria

(1) Observational studies (cohort studies, case-control studies, and cross-sectional studies) and experimental studies (randomized controlled trials and non-randomized controlled trials) of human consumption of PM, systematic reviews, and meta-analyses.

(2) Exposure factors include consumption of PM, outcomes include cancer, cardiovascular diseases, metabolic diseases, and other disease outcomes, as well as related health indicators.

(3) Reported effect measures include relative risk (RR), odds ratio (OR), or hazard ratio (HR) for qualitative studies; mean difference (MD) for quantitative studies.

(4) Studies including only healthy populations at baseline, excluding specific patient populations.

(5) No restrictions on gender, age, source of cases, duration of illness, country, region, or ethnicity.

#### Exclusion criteria

(1) Reviews or conference abstracts.

(2) Systematic reviews without meta-analysis.

(3) Systematic reviews or meta-analyses that did not consider PM consumption as an independent exposure factor or variable.

(4) Molecular, cellular, or animal experimental studies.

### Data extraction

Two assessors independently extracted the following information from each eligible systematic review and meta-analysis: health outcomes, first author's name, publication year, number of original studies, comparison type, type of original study design, effect model, number of participants and cases, types of pooled effect estimates (highest vs. lowest, high vs. low, low/medium/high, or additional per day/week), pooled effect estimates (OR, RR, or HR) with corresponding 95% CI, heterogeneity for each health outcome (I^2^ statistic and Cochrane Q test *p*-value), and publication bias (Egger's test *p*-value). For systematic reviews or meta-analyses examining the relationship between various dietary intake and disease outcomes or health risks, only data related to PM were extracted. Disputes between the two assessors regarding data extraction were resolved by a third researcher. Two assessors independently checked the study population and data source of included meta-analyses to avoid overlap; a corrected covered area (CCA) matrix was used to quantify the overlap of original studies among meta-analyses, and only the meta-analysis with higher methodological quality (AMSTAR2) was retained if the overlap rate exceeded 50%.

### Adherence to PROSPERO protocol

This umbrella review was prospectively registered in the International Prospective Register of Systematic Reviews (PROSPERO, registration number: CRD42022310748). The study was conducted in full adherence to the pre-specified protocol; no deviations were made in the study eligibility criteria, literature search strategy, data extraction items, or methodological quality assessment methods (AMSTAR2 and GRADE). All stratified analyses (e.g., by sex and geographical region) and comparative analyses (e.g., PRM vs. URM) were pre-specified in the original protocol.

### Methodological quality assessment and grading

The validated AMSTAR2 online quality assessment tool was used by two independent researchers to evaluate the methodological quality of each eligible article and assign an overall rating. AMSTAR2 is a reliable and effective methodological quality assessment tool with 16 items. Researchers assess the degree of adherence to each item using ratings of “Yes,” “Partial Yes,” or “No.” Items 2, 4, 7, 9, 11, 13, and 15 are key validity items that impact the methodological quality of systematic reviews/meta-analyses, while the others are non-critical. Based on the overall adherence to the items, the quality of the assessment is categorized into four levels: high, moderate, low, and very low.

Two researchers used the GRADE system to evaluate the quality of evidence. For each outcome in the included studies, we assessed whether to downgrade based on five factors: risk of bias, inconsistency, indirectness, imprecision, and publication bias. We considered upgrading based on three factors: dose-response relationship, large effect size, and negative bias. Ultimately, the confidence in the evidence was categorized into four levels: high, moderate, low, and very low.

## Results

### Literature search results

**Figure 1:**
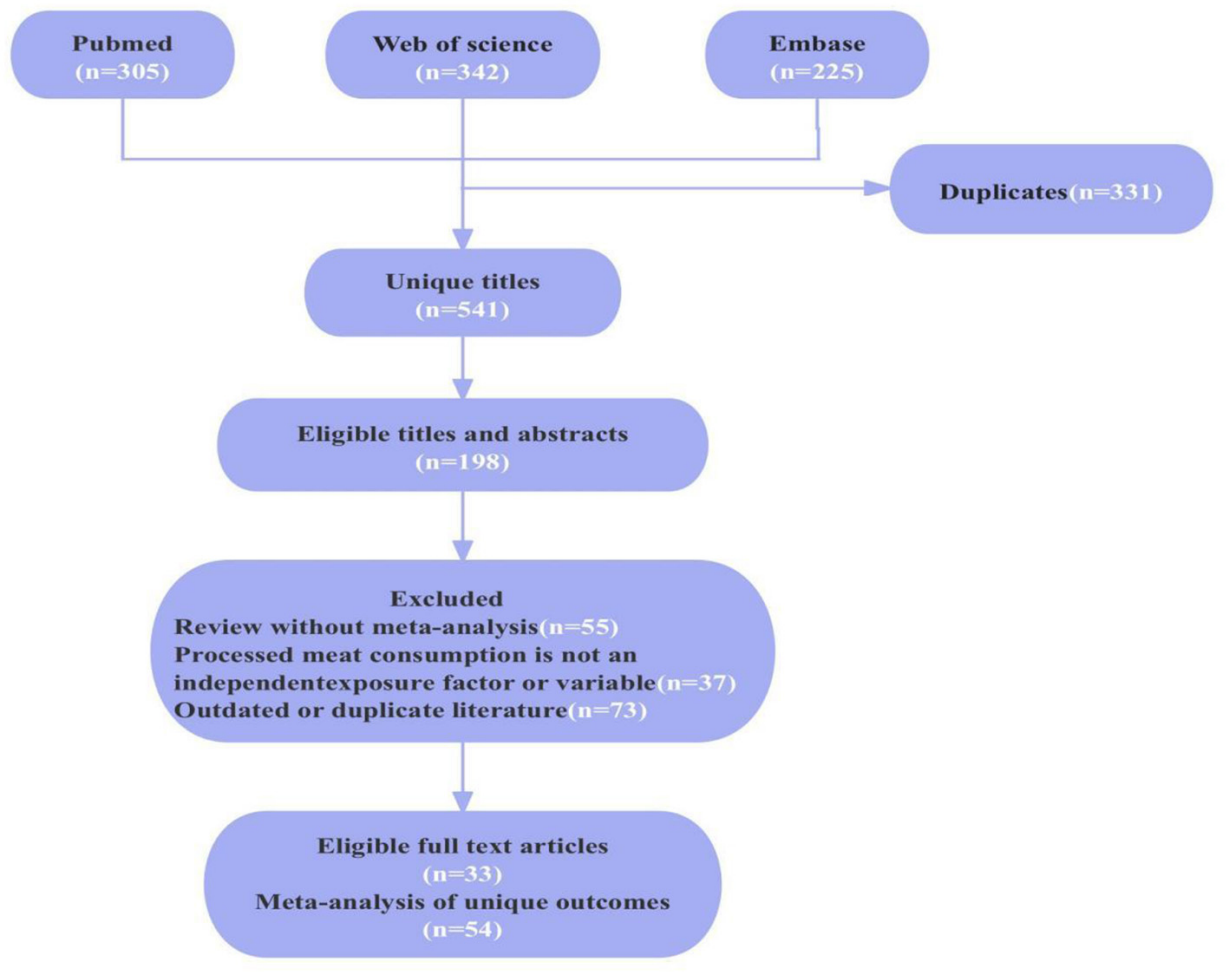
Flowchart of systematic search and selection process.

A total of 672 articles were obtained through searches in three databases, with specific numbers retrieved from each database as follows: Pubmed (*n* = 305), Embase (*n* = 342), and Web of Science (*n* = 255). After removing duplicates and carefully reviewing abstracts and full texts, 331 articles were excluded. Among them, 5 were narrative reviews, 50 were systematic reviews without meta-analysis, 37 were meta-analyses that did not consider PM consumption as an independent exposure factor or variable, and 73 were outdated or overlapping articles. Finally, 33 articles were included. The literature inclusion process is illustrated in Figure 1.

### All-cause mortality

A meta-analysis including 15 studies (*N* = 1,711,016) indicated (10) that the summary of highest vs. lowest intake (RR: 1.15; 95% CI: 1.10–1.21; GRADE: very low) showed significant heterogeneity among different studies (I^2^ = 78.5%, *P* < 0.001). Furthermore, compared to non-consumers of PM, consumers had a higher risk of mortality (RR: 1.05, 95% CI: 1.02–1.07; GRADE: very low; I^2^ = 11.2%; *P* = 0.342).

### Cancer mortality

A meta-analysis of 5 prospective cohort studies revealed a significant association between PM consumption and cancer mortality (RR: 1.08; 95% CI: 1.06–1.11; GRADE: very low). For each additional serving of PM per day, the risk of cancer mortality increased by 8% (13).

### Cardiovascular mortality and coronary heart disease mortality

A meta-analysis of 6 prospective cohort studies (*N* = 1,195,947) indicated that consuming one serving of PM per day was associated with a 15% increase in the risk of cardiovascular mortality (RR: 1.15; 95% CI: 1.07–1.24; GRADE: very low) (13). Evidence suggests a non-linear association between PM consumption and the risk of all-cause and cardiovascular mortality. There was no significant risk association between PM consumption and coronary heart disease mortality (RR: 0.92; 95% CI: 0.67–1.26;GRADE: very low) (14).

### Cancer outcomes

A meta-analysis of 19 case-control studies showed that compared to the lowest consumption, the highest consumption of PM increased the risk of esophageal cancer (RR: 1.50; 95% CI: 1.22–1.85; GRADE: very low). However, meta-analysis of cohort studies did not show a significant association between PM consumption and esophageal cancer risk. Dose-response analysis indicated a significant association between every 50 g/day increase in PM intake and esophageal cancer risk (RR: 1.41; 95% CI: 1.10–1.82; GRADE: low) (15). High consumption of PM was significantly associated with a 91% increase in the risk of oral and pharyngeal cancer (RR: 1.91; 95% CI: 1.19–3.06; GRADE: very low). Subgroup analysis revealed a significant correlation between high PM consumption and oral and pharyngeal cancer risk in South America (RR: 1.93; 95% CI: 1.25–3.00; GRADE: very low), but not in studies conducted in Europe (RR: 1.64; 95% CI: 0.59–4.60; GRADE: very low) and Asia (RR: 2.09; 95% CI: 0.70–6.29; GRADE: very low) (16). High consumption of PM was associated with a 57% increase in gastric cancer risk (RR: 1.57; 95% CI: 1.37–1.81; GRADE: very low). Dose-response analysis indicated that every 50 g/day increase in PM consumption was associated with a 72% higher risk of gastric cancer (RR: 1.72; 95% CI: 1.36–2.18; GRADE: very low). In stratified analysis, a significant association was observed in the male group (RR: 1.40; 95% CI: 1.13–1.74; GRADE: very low), but not in the female group. Significant correlations were found in all regions in the analysis based on geographical areas (Asian population RR: 1.74; 95% CI: 1.22–2.48; GRADE: very low; European population RR: 1.40; 95% CI: 1.14–1.73; GRADE: very low; North American population RR: 1.36; 95% CI: 1.15–1.61; GRADE: very low; Latin American population RR: 2.69; 95% CI: 1.76–4.12; GRADE: very low) (17). Compared to the lowest category, PM consumption was associated with a 9% increase in breast cancer risk (RR: 1.09; 95% CI: 1.03–1.16; GRADE: very low). Another study's dose-response analysis indicated a positive correlation between every 50 g/day increase in PM consumption and breast cancer incidence (RR: 1.18; 95% CI: 1.04–1.33; GRADE: very low) (18). Higher consumption of PM (RR: 1.20; 95% CI: 1.02–1.41; GRADE: very low) may increase the risk of hepatocellular carcinoma (19). Increased consumption of PM was associated with an increased risk of bladder cancer (RR: 1.16, 95% CI: 1.08–1.25; GRADE: very low) (20). Subgroup analysis showed statistical significance in case-control studies (RR: 1.21; 95% CI: 1.10–1.33; GRADE: very low), studies conducted in Europe/USA (RR: 1.12; 95% CI: 1.04–1.22; GRADE: very low), and other regions (RR: 1.39; 95% CI: 1.15–1.67; GRADE: very low). Studies published before 2009 had statistical significance (RR: 1.15; 95% CI: 1.02–1.29; GRADE: very low), as well as those published on or after 2009 (RR: 1.21; 95% CI: 1.09–1.34; GRADE: very low). One study (21) found that compared to low (< 30 g/week)/medium (30–60 g/week)/high (>60 g/week) levels of PM consumption, higher consumption was associated with an increased risk of nasopharyngeal cancer (GRADE: very low). Comparing the highest and lowest categories, PM consumption was significantly associated with prostate cancer (RR: 1.06, 95% CI: 1.01–1.10; GRADE: low). Linear dose-response analysis based on 12 prospective studies suggested that consuming an extra 50 g of PM per day might be associated with a 4% increase in overall prostate cancer risk (RR: 1.04; 95% CI: 1.00–1.08; GRADE: low) (22). Consumption of PM was significantly associated with renal cell carcinoma risk (RR: 1.13; 95% CI: 1.03–1.24; GRADE: very low) Renal cell carcinoma (23). PM consumption was significantly associated with colorectal cancer risk (RR: 1.14; 95% CI: 1.06–1.21; GRADE: low). Dose-response relationship analysis showed a significant association between every 50 g/day increase in PM consumption and colorectal cancer risk (RR: 1.17; 95% CI: 1.10–1.23; GRADE: low).

Additionally, no significant associations were detected between PM consumption and the risk of early-onset colorectal cancer (GRADE: very low) (24), pancreatic cancer (GRADE: very low) (25), glioma (GRADE: very low) (26), acute myeloid leukemia (GRADE: very low) (27), chronic lymphocytic leukemia/small lymphocytic lymphoma (GRADE: very low) (27), or ovarian cancer (GRADE: very low) (28).

### Metabolic outcomes

A meta-analysis based on 17 cohort studies revealed that the risk of type 2 diabetes mellitus (T2DM) was higher in individuals with high PM intake compared to those with low intake (RR: 1.25; 95% CI: 1.13–1.37; GRADE: very low) (29). Subgroup analysis indicated a consistent significant association in studies conducted in Europe and the United States, while no association was observed in studies conducted in Asia. Dose-response analysis showed a 46% increase in the risk of T2DM for every 50 g/day increase in PM intake. A non-linear dose-response relationship suggested a 30% increase in T2DM risk as intake increased to 30 g/day. Another meta-analysis based on 7 cohort studies found that high consumption of processed red meat increased the incidence of T2DM compared to the low intake group (RR: 1.27; 95% CI: 1.15–1.40; GRADE: low). A meta-analysis of 4 prospective cohort studies indicated a higher risk of metabolic syndrome associated with consumption of processed red meat (RR: 1.48; 95% CI: 1.11–1.97; GRADE: very low) (30). Consumption of PM was significantly associated with differences in body mass index (GRADE: very low) and waist circumference (GRADE: very low) (31). Based on meta-analysis of 3 cohort studies, consumption of PM was significantly associated with gestational diabetes risk (RR: 1.68; 95% CI: 1.38–2.05; GRADE: very low) (32).

### Comparisons of PM, processed red meat, and unprocessed red meat

This review further conducted comparative analyses of the associations between total PM, PRM, and URM and key health outcomes based on stratified data from the included meta-analyses, with all evidence rated as low or very low certainty by GRADE:

1. PRM vs. total PM: PRM showed slightly stronger positive associations with T2DM (PRM: RR: 1.27, 95% CI: 1.15–1.40; GRADE: low; total PM: RR: 1.25, 95% CI: 1.13–1.37; GRADE: low) and metabolic syndrome (PRM: RR: 1.48, 95% CI: 1.11–1.97; GRADE: very low; no significant association for total PM) (29, 30). No significant differences were observed in the associations of PRM and total PM with cardiovascular diseases and cancer outcomes.

2. PRM vs. URM: No significant associations were found between URM consumption and any assessed health outcomes (including cancers, cardiovascular diseases, and metabolic disorders). In contrast, PRM was significantly associated with an increased risk of T2DM, colorectal cancer, gastric cancer, and cardiovascular mortality (14, 17), indicating that adverse health effects are driven by the processing of red meat rather than red meat itself.

3. Processed beef vs. processed pork: Limited direct comparative data suggested that processed pork was associated with slightly higher risks of gastric cancer (RR:1.81, 95% CI:1.45–2.27; GRADE: very low) and colorectal cancer (RR:1.22, 95% CI:1.10–1.35; GRADE: very low) compared with processed beef (gastric cancer: RR:1.53, 95% CI:1.21–1.93; colorectal cancer: RR:1.15, 95% CI:1.06–1.25; GRADE: very low) (17, 33). However, the number of direct comparative studies was small, and heterogeneity was high (I^2^ > 70%) limiting the robustness of these findings.

### Cardiovascular outcomes

Comparing the highest and lowest categories, there is a positive correlation between PM intake and the risk of coronary heart disease (RR: 1.15; 95% CI: 0.99–1.33; GRADE: low). Dose-response analysis indicates a positive correlation between an increase of 50 g of PM per day and the risk of coronary heart disease (RR: 1.27; 95% CI: 1.09–1.49; GRADE: low) (34). High intake of PM, compared to the lowest category, is significantly associated with the risk of heart failure (RR: 1.23; 95% CI: 1.07–1.41; GRADE: very low). Stratified analysis by geographical location found significant associations only among Europeans, with no significant association among Americans (35). There is a positive correlation between PM intake and the risk of stroke (RR: 1.16; 95% CI: 1.07–1.26; GRADE: low). An increase of 50 g of PM per day is positively correlated with the risk of stroke (RR: 1.17; 95% CI: 1.02–1.34; GRADE: very low). As PM intake increases to >70 g/day, the risk of stroke increases by approximately 15% (34). The risk of hypertension is positively correlated with PM intake (RR: 1.12; 95% CI: 1.02–1.23; GRADE: very low), with every 50 g/day increase in PM consumption (RR: 1.12; 95% CI: 1.00–1.26; GRADE: very low). With an increase in PM intake of 30 g/day, the risk of hypertension increases by approximately 7%; beyond this value, there is no apparent additional risk increase (33). The risk of ischemic heart disease is positively associated with 50 g/day intake of PM (RR: 1.18; 95% CI: 1.12–1.25; GRADE: very low), and subgroup analysis suggests that this association is limited to studies conducted in the United States and Europe, with evidence of a negative correlation in the pooled analysis of two studies conducted in Asia (36).

### Other outcomes

Consumption of PM is significantly associated with the risk of kidney stones (RR: 1.29; 95% CI: 1.10–1.51; GRADE: very low) (37). A meta-analysis incorporating five cohort studies suggests that compared to the lowest category, high intake of processed red meat increases the risk of chronic obstructive pulmonary disease by 40% (HR: 1.40; 95% CI: 1.21–1.62; GRADE: low). Linear dose-response meta-analysis indicates that for every increase of 50 g/week in processed red meat intake, the risk of chronic obstructive pulmonary disease increases by 8% (HR: 1.08; 95% CI: 1.03–1.13; GRADE: very low). Evidence suggests a non-linear relationship between intake of processed red meat and the risk of chronic obstructive pulmonary disease (*P* < 0.001) (38). The intake of PM is significantly associated with the risk of cognitive decline (RR: 1.67; 95% CI: 1.46–1.92; GRADE: low), and dose-response analysis indicates a significant association between higher risk of cognitive decline and each additional 50 g/d of PM intake (RR: 1.12; 95% CI: 1.08–1.17; GRADE: low) (39). Furthermore, there is no significant association between PM consumption and the risk of Barrett's esophagus (OR: 1.03; 95% CI: 0.73–1.46; GRADE: very low).

### Heterogeneity of included studies

Among all included studies, approximately 30% had I^2^ < 25%; About 48% of the studies have moderate heterogeneity, i.e., 25% < I^2^ < 75%; About 17% of studies have I^2^ > 75%; Additionally, three studies did not report heterogeneity. The heterogeneity observed in this study suggests that the magnitude and mechanisms of health risks may vary due to differences in population, methodology, intake levels, and other factors. Future research should focus on standardizing study designs, clarifying definitions of health outcomes, refining classifications of PMs and their processing methods, and further exploring the roles of biological mechanisms such as genetics and gut microbiota. Additionally, randomized controlled trials and longitudinal studies could help clarify the causal relationship between PM consumption and health outcomes, providing more targeted guidance for public health policies.

### AMSTAR2 and GRADE classification of included studies

The methodological quality of the included systematic reviews was assessed using the AMSTAR2 scale, resulting in 13% rated as high, 1% as moderate, 43% as low, and 43% as critically low. This is mainly due to the majority of studies not reporting the funding sources of the included studies in the meta-analyses (item 10). Regarding GRADE ratings, 28% were classified as low, 72% as critically low, with no study outcomes rated as high or moderate. For detailed information, see Table 1.

**Table 1:** Assessment of AMSTAR2 scores and GRADE classification.

Outcomes	Year	First author	AMSTAR2	GRADE
Cancer Outcomes				
Early onset colorectal cancer	2023	Hua	Very low	Very low
Esophageal cancer	2019	Zhao	Very low	Very low
Pancreatic cancer	2022	Kim	Low	Very low
Glioma	2015	Saneei	Low	Very low
AML	2018	Sergentanis	Very low	Very low
CLL/SLL	2019	Sergentanis	Very low	Very low
Oral and pharyngeal cancer	2014	Jing Xu	Low	Very low
Gastric cancer	2019	Kim	High	Very low
Breast cancer	2018	Farvid	Low	Very low
Bladder cancer	2022	Yu	Low	Very low
Hepatocellular carcinoma	2022	Yu	Low	Very low
Prostatic cancer	2022	Nouri-Majd	High	Low
Nasopharyngeal carcinoma	2016	Li	Very low	Very low
Non-hodgkin's lymphoma	2015	Yang	Moderate	Low
Colorectal	2018	Schwingshackl	Low	Low
Renal cell carcinoma	2017	Zhang	Low	Very low
Cancer mortality	2016	Wang	Low	Very low
Cardiovascular outcomes				
Coronary heart disease mortality rate	2022	Medeiros	Very low	Very low
Cardiovascular mortality	2016	Wang	Low	Very low
Coronary heart disease	2019	Bechthold	High	Low
Apoplexy	2019	Bechthold	High	Low
Incidence rate of stroke	2023	Medeiros	Very low	Very low
Heart failure	2019	Cui	Low	Very low
Hypertension	2017	Schwingshackl	Very low	Very low
Ischemic heart disease	2021	Papier	High	Low
Metabolic outcomes				
T2DM	2021	Yang	Low	Very low
T2DM	2021	Zhang	Very low	Very low
Gestational diabetes	2021	Quan	Very low	Very low
Metabolic syndrome	2021	Guo	Very low	Very low
BMI	2014	Rouhani	Very low	Very low
WC	2014	Rouhani	Very low	Very low
Other outcomes				
BE	2016	Zhao	Very low	Very low
Kidney stone	2021	Farzaneh	Low	Very low
Cognitive decline	2022	Daniele	Very low	Low
COPD	2018	Asma	Low	Low
All-Cause Mortality	2022	Petek	Low	Very low
Dose-response relationship				
Pancreatic cancer	2022	Kim	Very low	Very low
Ovarian cancer	2011	A Wallin	Low	Very low
Renal cell carcinoma	2017	Zhang	Very low	Very low
Kidney stone	2021	Asoudeh	Low	Very low
Esophageal cancer	2020	Zhao	Very low	Low
Gastric cancer	2019	Kim	Low	Very low
Breast cancer	2020	Kazemi	Very low	Very low
Prostatic cancer	2022	Nouri-Majd	High	Low
Non-Hodgkin's lymphoma	2015	Yang	Low	Very low
Colorectal	2018	Schwingshackl	Low	Low
Ischemic heart disease	2021	Papier	High	Low
Coronary heart disease	2019	Bechthold	Low	Low
Apoplexy	2019	Bechthold	Low	Very low
Hypertension	2017	Schwingshackl	Very low	Very low
COPD	2018	Salari-Moghaddam	Low	Very low
Cognitive decline	2022	Wei	Very low	Low
T2DM	2021	Yang	Very low	Low

### Stratified analyses by sex and geographical region

To explore potential effect modification, we present findings from subgroup analyses based on sex and geographical region where such data were available in the included meta-analyses. The results are summarized in Table 2.

**Table 2:** Stratified analyses of the association between processed meat consumption and health outcomes by sex and geographical region.

Health outcome	Stratification variable	Subgroup	Summary estimate (95% CI)	Reference
Cancer outcomes				
Gastric cancer	Sex	Men	RR 1.40 (1.13–1.74)	Kim et al. (17)
		Women	Not significant	
	Region	Asia	RR 1.74 (1.22–2.48)	Kim et al. (17)
		Europe	RR 1.40 (1.14–1.73)	
		North America	RR 1.36 (1.15–1.61)	
		Latin America	RR 2.69 (1.76–4.12)	
Oral and pharyngeal cancer	Region	South America	RR 1.93 (1.25–3.00)	Xu et al. (16)
		Europe	RR 1.64 (0.59–4.60)	
		Asia	RR 2.09 (0.70–6.29)	
Bladder cancer	Region	Europe/USA	RR 1.12 (1.04–1.22)	Yu et al. (20)
		Other Regions	RR 1.39 (1.15–1.67)	
Cardiovascular outcomes				
Heart failure	Region	Europe	RR 1.23 (1.07–1.41)	Cui et al. (35)
		America	Not Significant	
Ischemic heart disease	Region	USA & Europe	RR 1.18 (1.12–1.25)	Papier et al. (36)
		Asia	Negative correlation (not significant)	
Metabolic outcomes				
Type 2 diabetes (T2DM)	Region	Europe & USA	Significant association	Yang et al. (29)
		Asia	No significant association	

### Sex-specific associations

A limited number of meta-analyses provided sex-stratified results. For gastric cancer, a significant association between high PM consumption and increased risk was observed in men (RR: 1.40; 95% CI: 1.13–1.74; GRADE: very low) but not in women (17). For the majority of other health outcomes, including colorectal cancer, breast cancer, and type 2 diabetes, the included meta-analyses either did not perform or did not report sex-stratified estimates, precluding a comprehensive analysis of sex-based differences.

### Region-specific associations

Notable geographical variations were observed for several health outcomes. The association between PM consumption and oral and pharyngeal cancer was significant in studies conducted in South America (RR: 1.93; 95% CI: 1.25–3.00; GRADE: very low) but was not statistically significant in studies from Europe or Asia (16). Conversely, the positive association with gastric cancer was consistent across all major geographical regions, including Asia, Europe, North America, and Latin America, with the highest risk observed in the Latin American population (GRADE: very low). For type 2 diabetes, a significant association was consistently found in studies from Europe and the United States (GRADE: very low), whereas no significant association was reported in the single study from Asia (29). Similarly, the risk of ischemic heart disease was positively associated with PM intake in studies from the United States and Europe (GRADE: low), but a pooled analysis of two studies from Asia suggested a non-significant, inverse association (36).

## Discussion

**Figure 2:**
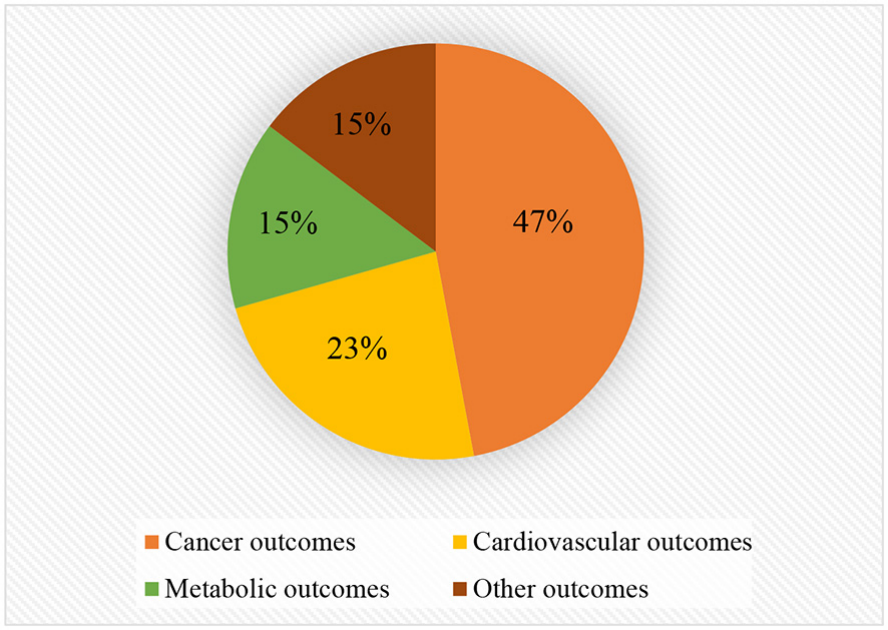
Map of outcomes associated with processed meat consumption.

In recent years, PM has been the subject of many meta-analyses concerning various health outcomes. This umbrella review incorporates a total of 54 meta-analyses, encompassing 37 health outcomes (For detailed information, see Table 3). The findings of this review indicate that consumption of PM is positively associated with the risk of diseases such as esophageal cancer, oral and pharyngeal cancer, gastric cancer, colorectal cancer, breast cancer, and metabolic syndrome. The association between PM consumption and cancer, cardiovascular, metabolic, and other outcomes is elaborated in Figure 2. The adverse health effects of PM may be attributed to the following mechanisms:

**Table 3:** Dose-response relationship between processed meat consumption and multiple health outcomes.

Outcome	Level of comparison	Exposure	MA metric	Estimate (95%CI)	No of studies (co/cc)	No of cases/total	I^2^; Q test *P* value	Egger test *P* value	Effects model
Esophageal cancer (15)	50 g/day increment	PM	RR	1.41 (1.10–1.82)	3 (3/0)	NA/NA	0%, 0.42	NA	R
Gastric cancer (17)	50 g/day increment	PM	RR	1.72 (1.36–2.18)	18 (7/11)	5,952/126,6661	72.1%, < 0.001	0.039	R
Breast cancer (40)	50 g/day increment	PM	RR	1.18 (1.04–1.33)	17 (15/2)	34,414/NA	63.5%, < 0.001	NA	R
Prostatic cancer (22)	50 g/day increment	PM	RR	1.06 (1.01–1.10)	13 (NA/NA)	NA/NA	1.5%, 0.43	0.06	NA
Non-Hodgkin lymphoma (41)	50 g/day increment	PM	RR	1.28 (1.08–1.53)	14 (3/11)	12,184/917,992	72.4%, < 0.001	NA	R
colorectal cancer (42)	50 g/day increment	PM	RR	1.17 (1.10–1.23)	18 (NA/NA)	20,283/NA	6%, 0.39	0.66	R
Ischemic heart disease (35)	50 g/day increment	PM	RR	1.18 (1.12–1.25)	10 (10/0)	31,426/NA	37.7%, 0.09	0.28	F
Coronary heart disease (34)	50 g/day increment	PM	RR	1.27 (1.09–1.49)	3 (NA/NA)	NA/NA	0%, 0.51	NA	R
Stroke (34)	50 g/day increment	PM	RR	1.17 (1.02–1.34)	6 (NA/NA)	9,492/NA	56%, 0.05	NA	R
Hypertension (33)	50 g/day increment	PM	RR	1.12 (1.00–1.26)	5 (NA/NA)	NA/NA	82%, < 0.001	NA	R
COPD (38)	50g/day increment	PRM	HR	1.08 (1.03–1.13)	5 (5/0)	8,338/28,9952	90.6%, < 0.001	NA	R
Cognitive decline (39)	50 g/day increment	PM	RR	1.12 (1.08–1.17)	NA(NA/NA)	NA/NA	NA	NA	R
T2DM (29)	50 g/day increment	PM	RR	1.46 (1.26–1.69)	NA(NA/NA)	NA/NA	NA	NA	R

RR, risk ratio; co, cohort study; cc, case-control study; RCT, randomized controlled tria; NRCT, non-randomized controlled trial; CI, confidence interval; R, random effects model; F, fixed effects model; NA, not available; PM, processed meat; COPD, chronic obstructive pulmonary disease; T2DM, type 2 diabetes mellitus.

During the processing, preservation, and high-temperature cooking of meat, carcinogenic substances such as heterocyclic amines (HCAs) (43) and polycyclic aromatic hydrocarbons (PAHs) are formed. To inhibit bacterial growth, nitrate and nitrite salts are added during the processing of meat products. Nitrite salts can undergo nitrosation reactions with amines and amides, forming carcinogenic N-nitroso compounds. Red meat and its processed products contain high levels of heme iron, and studies have shown that heme iron can catalyze and stimulate the formation of reactive oxygen species, inducing DNA damage and excessive proliferation (44); induce lipid peroxidation in food and intestines, producing lipid peroxides (45); and catalyze endogenous nitrosation (46), among other mechanisms, to exert carcinogenic effects. Furthermore, a recent meta-analysis incorporating 11 prospective studies (*N* = 323,788) indicated (47) that the higher the intake of heme iron, the higher the risk of type 2 diabetes (95% CI: 1.07, 1.35); dose-response analysis showed that for every 1 mg/d increase in heme iron intake, the risk of developing type 2 diabetes increased by 16% (95% CI: 1.03, 1.30). Another meta-analysis incorporating 19 prospective studies (*N* = 720,427) found that higher heme iron intake was associated with an increased risk of cardiovascular mortality (RR, 1.19; 95% CI: 1.01–1.39) (48).

Additionally, PM consumption has been associated with gut microbiota dysbiosis, characterized by a reduced abundance of beneficial commensal bacteria (e.g., *Bifidobacterium* and *Lactobacillus*) and an increase in pro-inflammatory microbial taxa. This dysbiosis induces chronic low-grade inflammation in the gastrointestinal tract, which is a key pathogenic factor for the development of colorectal cancer, metabolic syndrome, and cardiovascular diseases (2).

During processes like curing and smoking, meat is often infused with significant amounts of salt. Excessive consumption of PM products can result in an overabundance of sodium intake. Research has demonstrated that a high-salt diet is associated with increased risks of hypertension and cardiovascular diseases (49). An article published in the Journal of the American Medical Association conducted a prospective dietary crossover study involving 213 participants aged between 50 and 75 from February 2021 to April 2023. The findings revealed that compared to a high-sodium diet, a low-sodium diet led to a decrease in mean arterial pressure in 73.4% of individuals, with a mean systolic blood pressure difference of 8 mm Hg between high-sodium and low-sodium diets (50). Additionally, PMs contain both unsaturated and saturated fatty acids, with a higher content of saturated fatty acids. Excessive intake of saturated fatty acids increases the risk of obesity.

This study included two analyses on the association between PM consumption and T2DM risk, with one examining total PM as the exposure and the other focusing specifically on PRM. Several plausible biological mechanisms may explain the relationship between red meat consumption and the risk of T2DM, and the protein content of PRM is not the primary driver of this relationship (51): PRM contains saturated fatty acids and branched-chain amino acids, which can lead to insulin resistance and subsequently increase the risk of T2DM (52). N-nitroso compounds are toxic to pancreatic β-cells and impair insulin secretion in animal models (53). High levels of heme iron can promote the oxidation of free fatty acids and increase the levels of free radicals, thereby damaging pancreatic β cells (54).

To mitigate the adverse health risks associated with PM consumption, several alternative processing methods have been proposed based on current epidemiological and experimental evidence, which can reduce carcinogen formation and improve the nutritional profile of PMs: (1) Nitrite/nitrate reduction or replacement: Natural antioxidants (e.g., polyphenols from rosemary, green tea, and cranberries) can replace or reduce synthetic nitrites/nitrates; these antioxidants inhibit the formation of N-nitroso compounds by 30–60% while maintaining microbial safety and product shelf life (1). (2) Mild processing and cooking techniques: Adopting low-temperature processing (e.g., vacuum cooking, steaming, boiling) instead of high-temperature frying, grilling, or direct smoking significantly reduces the formation of heterocyclic amines (HCAs) and polycyclic aromatic hydrocarbons (PAHs) (43). (3) Low-sodium curing technologies: Partial substitution of sodium chloride (NaCl) with potassium chloride (KCl, 30–50%) combined with natural preservatives (e.g., lactic acid bacteria) lowers the sodium content of PMs without affecting flavor or microbial stability, thereby reducing hypertension risk (49, 50). (4) Microbial fermentation with selected starter cultures: Using specific Lactobacillus or Bifidobacterium strains for fermentation reduces lipid peroxidation and heme iron-induced oxidative stress in PMs, and further modulates the gut microbiota when consumed (45).

Although numerous studies have revealed the association between PM consumption and various health issues, there are still several limitations in the current body of research. First, many studies rely on self-reported dietary recall methods, which may introduce some bias. Second, most studies are observational, making it difficult to fully control for potential confounding factors such as dietary habits, genetic factors, and lifestyle. Additionally, the types, quantities, and processing methods of PMs vary, which could contribute to the heterogeneity of results. Therefore, caution is still needed when interpreting the relationship between PM consumption and health outcomes. Future research should focus on the long-term effects of PM on health and further explore its specific mechanisms. More randomized controlled trials and longitudinal cohort studies should be conducted to provide detailed classifications of PM types and processing methods, as well as their relationships with specific health outcomes. Moreover, with the advancement of precision medicine, exploring how PM affects different individuals, considering factors like genetic polymorphisms and variations in the gut microbiome, may offer more personalized recommendations for public health interventions.

### Advantages and limitations

This umbrella review summarizes the current associations between PM consumption and various health outcomes. AMSTAR 2 and GRADE were employed to assess the quality of methods and the evidence for each meta-analysis included. Some studies in the literature have investigated both red meat and PM together. This review focuses exclusively on the impact of PM consumption on health outcomes, providing a clearer understanding of the association between PM consumption and health outcomes. The review indicates that some health outcomes exhibit dose-response relationships, offering quantitative references for the association between PM consumption and various health outcomes. Most of the health outcomes discussed in this review is derived from observational studies. Due to heterogeneity and bias across studies, this may limit the strength and clarity of the associations between PM consumption and each health outcome. Physiological outcomes were omitted in this review, as its aim was to investigate the association between PM consumption and various health outcomes. Furthermore, most of the health outcomes were derived from observational studies, and heterogeneity and bias among studies may limit the association effects of each health outcome. A key limitation of this study is that it is a descriptive umbrella review with no quantitative re-analysis of the summary estimates from the included meta-analyses (e.g., random-effects models, prediction intervals) or re-analysis of individual primary study data, which limits the ability to further quantify association strength and reduce heterogeneity. The majority of included evidence was rated as low or very low quality by the GRADE system, meaning reported associations between PM and adverse health outcomes should be interpreted with extreme caution, and no definitive causal inferences can be drawn. Additionally, there is a lack of sufficient direct comparative studies between different PM subtypes (e.g., processed beef vs. pork) and between PRM and URM in the existing literature, leading to high heterogeneity in comparative analyses and limiting subtype-specific public health guidance. The articles included in this review mostly utilized food frequency questionnaires to assess PM intake, which may introduce limitations due to recall bias or inadequate memory, leading to inaccurate measurements of PM intake. Although some original studies reported results stratified by sex or region, the majority of the included meta-analyses only provided overall estimates. Where sex-specific or region-specific data were available in the original articles, they were not consistently extracted or synthesized in the meta-analyses we included. This limits our ability to draw conclusions about differential effects across demographic or geographic subgroups. Future umbrella reviews should endeavor to extract and synthesize stratified estimates where available. Additionally, a key limitation of this umbrella review pertains to stratified analyses. Although we identified and presented stratified data for sex and geographical region where available (as shown in Table 2), such analyses were not consistently performed or reported in the majority of the included meta-analyses. Consequently, for most health outcomes, we were unable to extract or synthesize sex-specific or region-specific estimates, even when the original observational studies might have contained such data. This limits our ability to draw definitive conclusions regarding potential effect modifications by these important demographic and geographic factors. Future systematic reviews and meta-analyses should prioritize the conduct and reporting of stratified analyses to address these gaps. As an umbrella review, our unit of analysis is the published systematic reviews and meta-analyses, not the individual primary studies they included. While we extracted the design of the primary studies during our data collection (as shown in Tables 4–8), many of the included meta-analyses themselves pooled results from different study types (e.g., combining cohort and case-control studies) and did not consistently provide subgroup results stratified by study design. Therefore, at the level of this umbrella review, it was not feasible to reclassify all primary studies across all health outcomes and perform a unified subgroup analysis while ensuring data integrity and accuracy. We acknowledge this as a limitation of our present study. For future research, we recommend conducting a systematic review and meta-analysis of individual participant data or primary studies, which would allow for a more direct and comprehensive subgroup analysis based on study design, thereby providing a more nuanced assessment of the strength of evidence. In addition, the relationship between PM consumption and various health outcomes (such as cancer, metabolic diseases, cardiovascular diseases, etc.) is complex and may be influenced by multiple factors (such as genetics, lifestyle, dietary factors, etc.). While umbrella reviews can summarize existing evidence, their results may be constrained when considering these confounding factors, making it difficult to draw in-depth causal inferences. Finally, the quality of the meta-analysis evidence included in this review was rated as low or very low, with no evidence rated as moderate or high quality. Future research with higher quality evidence is needed to confirm the association between PM consumption and various health outcomes. The conclusions of this study are based on low to very low certainty evidence by GRADE, and no definitive causal inferences or risk thresholds can be drawn; future research needs high-quality prospective cohort studies and randomized controlled trials to validate the associations.

**Table 4:** Association between processed meat consumption and mortality.

Outcome	Level of comparison	Exposure	MA metric	Estimate (95%CI)	No of studies (co/cc)	No of cases/total	I^2^; Q test *P* value	Egger test *P* value	Effects model
All-cause mortality (10)	highest vs. lowest	PM	RR	1.15 (1.10–1.21)	14 (14/0)	225,000/1,810,416	78.5%, < 0.001	0.176	R
Cancer mortality (13)	highest vs. lowest	PM	RR	1.08 (1.06–1.11)	5 (5/0)	45,738/1,144,264	0.0%, 0.450	0.54	R
Coronary heart disease mortality (14)	higher vs. lower	PM	RR	0.92 (0.67–1.26)	3 (3/0)	NA/NA	75%, 0.003	NA	R
Cardiovascular mortality (13)	highest vs. lowest	PM	RR	1.15 (1.07–1.24)	6(6/0)	332,781/1,195,947	75.4%, < 0.001	0.85	R

RR, risk ratio; co, cohort study; cc, case-control study; CI, confidence interval; R, random effects model; NA, not available; PM, processed meat.

**Table 5:** Association between processed meat consumption and cancer outcomes.

Outcome	Level of comparison	Exposure	MA metric	Estimate (95%CI)	No of studies (co/cc/ RCT/NRCT)	No of cases/total	I^2^; Q test *P* value	Egger test *P* value	Effects model
Early-onset colorectal cancer (24)	highest vs. lowest	PM	OR	1.26 (0.95–1.66)	3 (0/3/0/0)	NA/NA	59%, 0.09	NA	R
Esophageal cancer (15)	highest vs. lowest	PM	RR	1.21 (0.78–1.88)	3 (3/0/0/0)	NA/NA	66%, 0.01	NA	R
Pancreatic cancer (25)	highest vs. lowest	PM	RR	1.00 (0.85–1.18)	14 (14/0/0/0)	6,049/2,140,638	59.90%, 0.001	0.5	R
Glioma (26)	highest vs. lowest	PM	RR	1.14 (0.98–1.33)	17 (3/14/0/0)	5,259/820,860	50.6%, 0.01	0.07	R
AML (27)	highest vs. lowest	PM	RR	0.83 (0.64–1.08)	3 (3/0/0/0)	573/1,003,709	0.0%, 0.914	NA	R
CLL/SLL (27)	highest vs. lowest	PM	RR	0.94 (0.77–1.16)	3 (3/0/0/0)	1,520/1 038 528	19.8%, 0.288	NA	R
Esophageal cancer (15)	highest vs. lowest	PM	RR	1.50 (1.22–1.85)	19 (0/19/0/0)	NA/NA	54%, 0.003	NA	R
Oral and pharyngeal cancer (16)	highest vs. lowest	PM	RR	1.91 (1.19–3.06)	9 (0/9/0/0)	4,104/501,730	85.9%, < 0.001	0.999	R
Gastric cancer (17)	highest vs. lowest	PM	RR	1.57 (1.37–1.81)	28 (8/20/0/0)	10,645/NA	55.5%, < 0.001	0.048	R
Breast cancer (18)	highest vs. lowest	PM	RR	1.09 (1.03–1.16)	15 (2/11/1/1)	37,070/1,254,452	44.4%, 0.033	0.67	R
Bladder cancer (20)	highest vs. lowest	PM	RR	1.16 (1.08–1.25)	19 (6/13/0/0)	NA/NA	28.00%, 0.125	0.981	F
Hepatocellular carcinoma (19)	highest vs. lowest	PM	RR	1.20 (1.02–1.41)	7 (5/2/0/0)	NA/NA	26.3%, 0.228	0.615	F
Prostatic cancer (22)	highest vs. lowest	PM	RR	1.06 (1.01–1.10)	13 (NA/NA/0/0)	NA/NA	1.5%, 0.43	0.06	F
Nasopharyngeal carcinoma (21)	low/medium/high	PM	RR	1.46 (1.31–1.64)	11 (0/11/0/0)	4,278/4,534	61%, 0.004	0.57	R
Non-Hodgkin lymphoma (41)	highest vs. lowest	PM	RR	1.17 (1.07–1.29)	16 (3/13/0/0)	12,184/917,992	37.1%, 0.057	0.181	R
Colorectal cancer (42)	high vs. low	PM	RR	1.14 (1.06–1.21)	18(NA/NA/0/0)	20,283/NA	22%, 0.20	0.78	R
Renal cell carcinoma (23)	highest vs. lowest	PM	RR	1.13 (1.03–1.24)	19(4/15/0/0)	14,701/1,784,140	45.6%, 0.014	0.145	R

RR, risk ratio; OR, odds ratio; co, cohort study; cc, case-control study; RCT, randomized controlled tria; NRCT, non-randomized controlled trial; CI, confidence interval; R, random effects model; F, fixed effects model; NA, not available; PM, processed meat; AML, acute myeloid leukemia; CLL/SLL, chronic lymphocytic leukemia/small lymphocytic lymphoma.

**Table 6:** Association between processed meat consumption and cardiovascular outcomes.

Outcome	Level of comparison	Exposure	MA metric	Estimate (95%CI)	No of studies (co/cc)	No of cases/total	I^2^; Q test *P* value	Egger test *P* value	Effects model
Coronary heart disease (34)	highest vs. lowest	PM	RR	1.27 (1.09–1.49)	5 (NA/NA)	7,038/NA	44%, 0.09	0.38	R
Stroke (34)	highest vs. lowest	PM	RR	1.17 (1.02–1.34)	6 (NA/NA)	9,492/NA	12%, 0.43	0.34	R
Stroke (14)	higher vs. low	PM	RR	1.17 (1.08–1.26)	3 (3/0)	NA/NA	19%, 0.29	NA	R
Heart failure (35)	highest vs. lowest	PM	RR	1.23 (1.07–1.41)	5 (5/0)	8,426/113,743	58.9%, 0.045	0.102	R
Hypertension (33)	highest vs. lowest	PM	RR	1.12 (1.02–1.23)	5 (NA/NA)	97,441/NA	81%, < 0.001	NA	R
Ischemic heart disease (36)	highest vs. lowest	PM	RR	1.11 (1.06–1.16)	11 (11/0)	NA/NA	43.9%, 0.05	0.51	F

RR, risk ratio; co, cohort study; cc, case-control study; CI, confidence interval; R, random effects model; F, fixed effects model; NA, not available; PM, processed meat.

**Table 7:** Association between processed meat consumption and metabolic outcomes.

Outcome	Level of comparison	Exposure	MA metric	Estimate (95%CI)	No of studies (co/cc)	No of cases/total	I^2^; Q test *P* value	Egger test *P* value	Effects model
T2DM (29)	highest vs. lowest	PM	RR	1.25 (1.13–1.37)	17 (17/0)	NA/NA	92.1%, < 0.001	0.002	R
T2DM (29)	highest vs. lowest	PRM	RR	1.27 (1.15–1.40)	7 (7/0)	NA/465,995	81%, < 0.001	0.76	R
Gestational diabetes (32)	high vs. low	PRM	RR	1.68 (1.38–2.05)	3 (3/0)	NA/NA	74%, 0.61	NA	R
Metabolic syndrome (55)	highest vs. lowest	PRM	RR	1.48 (1.11–1.97)	4 (4/0)	NA/NA	64.7%, 0.037	0.259	R
BMI (31)	high vs. low	PM	MD	1.32 (0.64–2.00)	7 (NA/NA)	NA/NA	90.5%, < 0.001	0.27	R
WC (31)	high vs. low	PM	MD	2.77 (1.87–2.66)	2 (NA/NA)	NA/NA	0.0%, 0.463	0.052	R

RR, risk ratio; MD, mean difference; co, cohort study; cc, case-control study; CI, confidence interval; R, random effects model; NA, not available; PM, processed meat; PRM, processed red meat; T2DM, type 2 diabetes mellitus; BMI, Body Mass Index; WC, waist circumference.

**Table 8:** Association between processed meat consumption and other outcomes.

Outcome	Level of comparison	Exposure	MA metric	Estimate (95%CI)	No of studies (co/cc)	No of cases/total	I^2^; Q test *P* value	Egger test *P* value	Effects model
BE (56)	highest vs. lowest	PM	OR	0.85 (0.73–1.46)	3 (1/2)	NA/NA	27%, 0.25	0.86	R
Kidney stones (37)	highest vs. lowest	PM	RR	1.29 (1.10–1.51)	2 (2/0)	NA/NA	0.0%, 0.48	NA	F
Cognitive decline (39)	highest vs. lowest	PM	RR	1.67 (1.46–1.92)	4 (4/0)	NA/NA	0%, < 0.01	NA	R
COPD (38)	highest vs. lowest	PRM	HR	1.4 (1.21–1.62)	5 (5/0)	8,338/289,952	41.8%, 0.143	0.33	R

RR, risk ratio; OR, odds ratio; HR, hazard ratio; co, cohort study; cc, case-control study; CI, confidence interval; R, random effects model; F, fixed effects model; NA, not available; PM, processed meat; PRM, processed red meat; BE, Barrett's esophagus; COPD, chronic obstructive pulmonary disease.

## Conclusion

The key dose-response results reported below were selected based on their high public health relevance and stable effect estimates across the included meta-analyses. This descriptive umbrella review suggests that PM consumption is associated with an increased risk of various health outcomes, including various cancers, cardiovascular diseases, and metabolic disorders. PRM shows stronger and more consistent adverse associations than total PM, while no significant health risks are associated with URM, confirming that meat processing is the primary driver of these adverse effects. All identified associations are based on low to very low certainty evidence as assessed by the GRADE system, and consistent with the recommendations from the International Agency for Research on Cancer (IARC) and WCRF/AICR (2018), no level of PM intake can be confidently considered safe for chronic disease prevention. However, due to the limitations in the design and methodology of existing studies, more high-quality research is needed to further validate these relationships and explore their underlying mechanisms in greater depth.

## Data Availability

The original contributions presented in the study are included in the article/supplementary material, further inquiries can be directed to the corresponding author.

## References

[1] Bouvard V Loomis D Guyton KZ Grosse Y Ghissassi FE Benbrahim-Tallaa L . Carcinogenicity of consumption of red and processed meat. Lancet Oncol. (2015) 16:1599–600. doi: 10.1016/S1470-2045(15)00444-1 26514947

[2] Wolk A. Potential health hazards of eating red meat. J Intern Med. (2017) 281:106–22. doi: 10.1111/joim.12543 27597529

[3] Walker P Rhubart-Berg P McKenzie S Kelling K Lawrence RS. Public health implications of meat production and consumption. Public Health Nutr. (2005) 8:348–56. doi: 10.1079/PHN2005727 15975179

[4] Miller V Reedy J Cudhea F Zhang J Shi P Erndt-Marino J . Global, regional, and national consumption of animal-source foods between 1990 and 2018: findings from the global dietary database. Lancet Planet Health. (2022) 6:e243–56. doi: 10.1016/S2542-5196(21)00352-1 35278390 PMC8926870

[5] Zeng L Ruan M Liu J Wilde P Naumova EN Mozaffarian D . Trends in processed meat, unprocessed red meat, poultry, and fish consumption in the United States, 1999–2016. J Acad Nutr Diet. (2019) 119:1085–98.e12. doi: 10.1016/j.jand.2019.04.004 31234969 PMC6689198

[6] Farchi S De Sario M Lapucci E Davoli M Michelozzi P. Meat consumption reduction in Italian regions: health co-benefits and decreases in GHG emissions. PLoS ONE. (2017) 12:e0182960. doi: 10.1371/journal.pone.0182960 28813467 PMC5557600

[7] Kim K Hyeon J Lee SA Kwon SO Lee H Keum N . Role of total, red, processed, and white meat consumption in stroke incidence and mortality: a systematic review and meta-analysis of prospective cohort studies. J Am Heart Assoc. (2017) 6:e005983. doi: 10.1161/JAHA.117.005983 28855166 PMC5634267

[8] Diallo A Deschasaux M Latino-Martel P Hercberg S Galan P Fassier P . Red and processed meat intake and cancer risk: results from the prospective NutriNet-Santé cohort study. Int J Cancer. (2018) 142:230–7. doi: 10.1002/ijc.31046 28913916

[9] Pouchieu C Deschasaux M Hercberg S Druesne-Pecollo N Latino-Martel P Touvier M . Prospective association between red and processed meat intakes and breast cancer risk: modulation by an antioxidant supplementation in the SU.VI.MAX randomized controlled trial. Int J Epidemiol. (2014) 43:1583–92. doi: 10.1093/ije/dyu134 24994839

[10] Taneri PE Wehrli F Roa-Díaz ZM Itodo OA Salvador D Raeisi-Dehkordi H . Association between ultra-processed food intake and all-cause mortality: a systematic review and meta-analysis. Am J Epidemiol. (2022) 191:1323–35. doi: 10.1093/aje/kwac039 35231930

[11] Männistö S Kontto J Kataja-Tuomola M Albanes D Virtamo J. High processed meat consumption is a risk factor of type 2 diabetes in the alpha-tocopherol, beta-carotene cancer prevention study. Br J Nutr. (2010) 103:1817–22. doi: 10.1017/S0007114510000073 20187985 PMC3496924

[12] Johnston BC Zeraatkar D Han MA Vernooij RWM Valli C El Dib R . Unprocessed red meat and processed meat consumption: dietary guideline recommendations from the nutritional recommendations (NutriRECS) consortium. Ann Intern Med. (2019) 171:756–64. doi: 10.7326/M19-1621 31569235

[13] Wang X Lin X Ouyang YY Liu J Zhao G Pan A . Red and processed meat consumption and mortality: dose–response meta-analysis of prospective cohort studies. Public Health Nutr. (2016) 19:893–905. doi: 10.1017/S1368980015002062 26143683 PMC10270853

[14] De Medeiros GCBS Mesquita GXB Lima SCVC de Oliveira Silva DF De Azevedo KPM Pimenta IDSF . Associations of the consumption of unprocessed red meat and processed meat with the incidence of cardiovascular disease and mortality, and the dose-response relationship: a systematic review and meta-analysis of cohort studies. Crit Rev Food Sci Nutr. (2023) 63:1–14. doi: 10.1080/10408398.2022.2058461 35491892

[15] Zhao Z Wang F Chen D Zhang C. Red and processed meat consumption and esophageal cancer risk: a systematic review and meta-analysis. Clin Transl Oncol. (2020) 22:532–45. doi: 10.1007/s12094-019-02157-0 31270670

[16] Xu J Yang X Wu Y Li X Bai B. Meat consumption and risk of oral cavity and oropharynx cancer: a meta-analysis of observational studies. PLoS ONE. (2014) 9:e95048. doi: 10.1371/journal.pone.0095048 24736706 PMC3988178

[17] Kim SR Kim K Lee SA Kwon SO Lee J-K Keum N . Effect of red, processed, and white meat consumption on the risk of gastric cancer: an overall and dose–response meta-analysis. Nutrients. (2019) 11:826. doi: 10.3390/nu11040826 30979076 PMC6520977

[18] Farvid MS Stern MC Norat T Sasazuki S Vineis P Weijenberg MP . Consumption of red and processed meat and breast cancer incidence: a systematic review and meta-analysis of prospective studies. Int J Cancer. (2018) 143:2787–99. doi: 10.1002/ijc.31848 30183083 PMC8985652

[19] Yu J Liu Z Liang D Li J Ma S Wang G . Meat intake and the risk of hepatocellular carcinoma: a meta-analysis of observational studies. Nutr Cancer. (2022) 74:3340–50. doi: 10.1080/01635581.2022.2077386 35583453

[20] Yu J Li H Liu Z Wang T Zhou F Ma S . Meat intake and the risk of bladder cancer: a systematic review and meta-analysis of observational studies. Nutr Cancer. (2023) 75:825–45. doi: 10.1080/01635581.2022.2159043 36537666

[21] Li F Duan F Zhao X Song C Cui S Dai L . Red meat and processed meat consumption and nasopharyngeal carcinoma risk: a dose-response meta-analysis of observational studies. Nutr Cancer. (2016) 68:1034–43. doi: 10.1080/01635581.2016.1192200 27367552

[22] Nouri-Majd S Salari-Moghaddam A Aminianfar A Larijani B Esmaillzadeh A. Association between red and processed meat consumption and risk of prostate cancer: a systematic review and meta-analysis. Front Nutr. (2022) 9:801722. doi: 10.3389/fnut.2022.801722 35198587 PMC8859108

[23] Zhang S Wang Q He J. Intake of red and processed meat and risk of renal cell carcinoma: a meta-analysis of observational studies. Oncotarget. (2017) 8:77942–56. doi: 10.18632/oncotarget.18549 29100437 PMC5652826

[24] Hua H Jiang Q Sun P Xu X. Risk factors for early-onset colorectal cancer: systematic review and meta-analysis. Front Oncol. (2023) 13:1132306. doi: 10.3389/fonc.2023.1132306 37213277 PMC10196487

[25] Kim Y. The association between red, processed and white meat consumption and risk of pancreatic cancer: a meta-analysis of prospective cohort studies. Cancer Causes Control. (2023) 34:569–81. doi: 10.1007/s10552-023-01698-8 37071321

[26] Esmaillzadeh A Saneei P Willett W. Red and processed meat consumption and risk of glioma in adults: a systematic review and meta-analysis of observational studies. J Res Med Sci. (2015) 20:602. doi: 10.4103/1735-1995.165970 26600837 PMC4621656

[27] Sergentanis TN Ntanasis-Stathopoulos I Tzanninis I-G Gavriatopoulou M Sergentanis IN Dimopoulos MA . Meat, fish, dairy products and risk of hematological malignancies in adults – a systematic review and meta-analysis of prospective studies. Leuk Lymphoma. (2019) 60:1978–90. doi: 10.1080/10428194.2018.1563693 30912696

[28] Wallin A Orsini N Wolk A. Red and processed meat consumption and risk of ovarian cancer: a dose-response meta-analysis of prospective studies. Br J Cancer. (2011) 104:1196–201. doi: 10.1038/bjc.2011.49 21343939 PMC3068494

[29] Yang X Li Y Wang C Mao Z Zhou W Zhang L . Meat and fish intake and type 2 diabetes: dose–response meta-analysis of prospective cohort studies. Diabet Metab. (2020) 46:345–52. doi: 10.1016/j.diabet.2020.03.004 32302686

[30] Zhang R Fu J Moore JB Stoner L Li R. Processed and unprocessed red meat consumption and risk for type 2 diabetes mellitus: an updated meta-analysis of cohort studies. Int J Environ Res Public Health. (2021) 18:10788. doi: 10.3390/ijerph182010788 34682532 PMC8536052

[31] Rouhani MH Salehi-Abargouei A Surkan PJ Azadbakht L. Is there a relationship between red or processed meat intake and obesity? A systematic review and meta-analysis of observational studies. Obes Rev. (2014) 15:740–8. doi: 10.1111/obr.12172 24815945

[32] Quan W Zeng M Jiao Y Li Y Xue C Liu G . Western dietary patterns, foods, and risk of gestational diabetes mellitus: a systematic review andmeta-analysis of prospective cohort studies. Adv Nutr. (2021) 12:1353–64. doi: 10.1093/advances/nmaa184 33578428 PMC8321835

[33] Schwingshackl L Schwedhelm C Hoffmann G Knüppel S Iqbal K Andriolo V . Food groups and risk of hypertension: a systematic review and dose-response meta-analysis of prospective studies. Adv Nutr. (2017) 8:793–803. doi: 10.3945/an.117.017178 29141965 PMC5683007

[34] Bechthold A Boeing H Schwedhelm C Hoffmann G Knüppel S Iqbal K . Food groups and risk of coronary heart disease, stroke and heart failure: a systematic review and dose-response meta-analysis of prospective studies. Crit Rev Food Sci Nutr. (2019) 59:1071–90. doi: 10.1080/10408398.2017.1392288 29039970

[35] Cui K Liu Y Zhu L Mei X Jin P Luo Y . Association between intake of red and processed meat and the risk of heart failure: a meta-analysis. BMC Public Health. (2019) 19:354. doi: 10.1186/s12889-019-6653-0 30922287 PMC6440157

[36] Papier K Knuppel A Syam N Jebb SA Key TJ. Meat consumption and risk of ischemic heart disease: a systematic review and meta-analysis. Crit Rev Food Sci Nutr. (2023) 63:426–37. doi: 10.1080/10408398.2021.1949575 34284672

[37] Asoudeh F Talebi S Jayedi A Marx W Najafi MT Mohammadi H . Associations of total protein or animal protein intake and animal protein sources with risk of kidney stones: a systematic review and dose–response meta-analysis. Adv Nutr. (2022) 13:821–32. doi: 10.1093/advances/nmac013 35179185 PMC9156392

[38] Salari-Moghaddam A Milajerdi A Larijani B Esmaillzadeh A. Processed red meat intake and risk of COPD: a systematic review and Q7 dose-response meta-analysis of prospective cohort studies. Clin Nutr. (2019) 38:1109–16. doi: 10.1016/j.clnu.2018.05.020 29909249

[39] Quan W Xu Y Luo J Zeng M He Z Shen Q . Association of dietary meat consumption habits with neurodegenerative cognitive impairment: an updated systematic review and dose–response meta-analysis of 24 prospective cohort studies. Food Funct. (2022) 13:12590–601. doi: 10.1039/D2FO03168J 36385382

[40] Kazemi A Barati-Boldaji R Soltani S Mohammadipoor N Esmaeilinezhad Z Clark CCT . Intake of various food groups and risk of breast cancer: a systematic review and dose-response meta-analysis of prospective studies. Adv Nutr. (2021) 12:809–49. doi: 10.1093/advances/nmaa147 33271590 PMC8166564

[41] Yang L Dong J Jiang S Shi W Xu X Huang H . Red and processed meat consumption increases risk for non-hodgkin lymphoma a prisma-compliant meta-analysis of observational studies. Medicine. (2015) 94:e1729. doi: 10.1097/MD.0000000000001729 26559248 PMC4912242

[42] Schwingshackl L Schwedhelm C Hoffmann G Knüppel S Laure Preterre A Iqbal K . Food groups and risk of colorectal cancer. Int J Cancer. (2018) 142:1748–58. doi: 10.1002/ijc.31198 29210053

[43] Sinha R. An epidemiologic approach to studying heterocyclic amines. Mutat Res. (2002) 506–507:197–204. doi: 10.1016/S0027-5107(02)00166-5 12351159

[44] Ishikawa S Tamaki S Ohata M Arihara K Itoh M. Heme induces DNA damage and hyperproliferation of colonic epithelial cells via hydrogen peroxide produced by heme oxygenase: a possible mechanism of heme-induced colon cancer. Mol Nutr Food Res. (2010) 54:1182–91. doi: 10.1002/mnfr.200900348 20112302

[45] Pierre F Tache S Guéraud F Rerole AL Jourdan M-L Petit C. Apc mutation induces resistance of colonic cells to lipoperoxide-triggered apoptosis induced by faecal water from haem-fed rats. Carcinogenesis. (2007) 28:321–7. doi: 10.1093/carcin/bgl127 16885197

[46] Cross AJ Pollock JRA Bingham SA. Haem, not protein or inorganic iron, is responsible for endogenous intestinal N-nitrosation arising from red meat. Cancer Res. (2003) 63:2358–60. doi: 10.1016/s0027-5107(02)00166-5 12750250

[47] Shahinfar H Jayedi A Shab-Bidar S. Dietary iron intake and the risk of type 2 diabetes: a systematic review and dose-response meta-analysis of prospective cohort studies. Eur J Nutr. (2022) 61:2279–96. doi: 10.1007/s00394-022-02813-2 35107626

[48] Han M Guan L Ren Y Zhao Y Liu D Zhang D . Dietary iron intake and risk of death due to cardiovascular diseases: a systematic review and dose-response meta-analysis of prospective cohort studies. Asia Pac J Clin Nutr. (2020) 29:309–21. doi: 10.6133/apjcn.202007_29(2).0014 32674239

[49] Strazzullo P D'Elia L Kandala N-B Cappuccio FP. Salt intake, stroke, and cardiovascular disease: meta-analysis of prospective studies. BMJ. (2009) 339:b4567. doi: 10.1136/bmj.b4567 19934192 PMC2782060

[50] Gupta DK Lewis CE Varady KA Su YR Madhur MS Lackland DT . Effect of dietary sodium on blood pressure: a crossover trial. JAMA. (2023) 330:2258–66. doi: 10.1001/jama.2023.23651 37950918 PMC10640704

[51] Ericson U Sonestedt E Gullberg B Hellstrand S Hindy G Wirfält E . High intakes of protein and processed meat associate with increased incidence of type 2 diabetes. Br J Nutr. (2013) 109:1143–53. doi: 10.1017/S0007114512003017 22850191

[52] Newgard CB An J Bain JR Muehlbauer MJ Stevens RD Lien LF . A branched-chain amino acid-related metabolic signature that differentiates obese and lean humans and contributes to insulin resistance. Cell Metab. (2009) 9:311–26. doi: 10.1016/j.cmet.2009.02.002 19356713 PMC3640280

[53] Pan A Sun Q Bernstein AM Schulze MB Manson JE Willett WC . Red meat consumption and risk of type 2 diabetes: 3 cohorts of US adults and an updated meta-analysis. Am J Clin Nutr. (2011) 94:1088–96. doi: 10.3945/ajcn.111.018978 21831992 PMC3173026

[54] InterAct Consortium Bendinelli B Palli D Masala G Sharp SJ Schulze MB . Association between dietary meat consumption and incident type 2 diabetes: the EPIC-InterAct study. Diabetologia. (2013) 56:47–59. doi: 10.1007/s00125-012-2718-7 22983636

[55] Guo H Ding J Liang J Zhang Y. Association of red meat and poultry consumption with the risk of metabolic syndrome: a meta-analysis of prospective cohort studies. Front Nutr. (2021) 8:691848. doi: 10.3389/fnut.2021.691848 34307439 PMC8295459

[56] Zhao Z Pu Z Yin Z Yu P Hao Y Wang Q . Dietary fruit, vegetable, fat, and red and processed meat intakes and Barrett's esophagus risk: a systematic review and meta-analysis. Sci Rep. (2016) 6:27334. doi: 10.1038/srep27334 27256629 PMC4891687

